# Triglyceride-glucose index is associated with myocardial ischemia and poor prognosis in patients with ischemia and no obstructive coronary artery disease

**DOI:** 10.1186/s12933-024-02230-1

**Published:** 2024-05-31

**Authors:** Wen Zhang, Lu Liu, Guoqing Yin, Abdul-Quddus Mohammed, Lanqing Xiang, Xian Lv, Tingting Shi, Jassur Galip, Chunyue Wang, Ayman A. Mohammed, Redhwan M. Mareai, Fei Yu, Fuad A. Abdu, Wenliang Che

**Affiliations:** 1 Department of Cardiology, Shanghai Tenth People’s Hospital, Tongji University School of Medicine, 301 Yanchang Road, Shanghai, 200072 China; 2 Department of Nuclear Medicine, Shanghai Tenth People’s Hospital, Tongji University School of Medicine, Shanghai, China; 3 Department of Cardiology, Shanghai Tenth People’s Hospital Chongming branch, Shanghai, China

**Keywords:** INOCA, Myocardial perfusion imaging, Triglyceride-glucose index, Prognosis

## Abstract

**Background:**

Ischemia and no obstructive coronary artery disease (INOCA) is increasingly recognized and associated with poor outcomes. The triglyceride-glucose (TyG) index is a reliable alternative measure of insulin resistance significantly linked to cardiovascular disease and adverse prognosis. We investigated the association between the TyG index and myocardial ischemia and the prognosis in INOCA patients.

**Methods:**

INOCA patients who underwent both coronary angiography and myocardial perfusion imaging (MPI) were included consecutively. All participants were divided into three groups according to TyG tertiles (T1, T2, and T3). Abnormal MPI for myocardial ischemia in individual coronary territories was defined as summed stress score (SSS) ≥ 4 and summed difference score (SDS) ≥ 2. SSS refers to the sum of all defects in the stress images, and SDS is the difference of the sum of all defects between the rest images and stress images. All patients were followed up for major adverse cardiac events (MACE).

**Results:**

Among 332 INOCA patients, 113 (34.0%) had abnormal MPI. Patients with higher TyG index had a higher rate of abnormal MPI (25.5% vs. 32.4% vs. 44.1%; *p* = 0.012). Multivariate logistic analysis showed that a high TyG index was significantly correlated with abnormal MPI in INOCA patients (OR, 1.901; 95% CI, 1.045–3.458; *P* = 0.035). During the median 35 months of follow-up, 83 (25%) MACE were recorded, and a higher incidence of MACE was observed in the T3 group (T3 vs. T2 vs. T1: 36.9% vs. 21.6% vs. 16.4%, respectively; *p* = 0.001). In multivariate Cox regression analysis, the T3 group was significantly associated with the risk of MACE compared to the T1 group (HR, 2.338; 95% CI 1.253–4.364, *P* = 0.008).

**Conclusion:**

This study indicates for the first time that the TyG index is significantly associated with myocardial ischemia and poor prognosis among INOCA patients.

## Background

Symptoms and signs of myocardial ischemia have multifactorial factors, with obstructive coronary artery disease (CAD) being the predominant and widely recognized cause [1, 2]. However, approximately up to half of symptomatic patients are found to have non-obstructive CAD on invasive coronary angiography (CAG) [3, 4]. This chronic coronary syndrome, termed ischemia and no obstructive CAD (INOCA), is increasingly acknowledged as a significant contributor to major adverse cardiovascular events (MACE) [5, 6]. INOCA presents an intermediate prognosis when compared to obstructive CAD, with the highest risk of MACE [7]. However, it is important to note that the MACE risk of INOCA is higher than individuals with minimal or no atherosclerosis [5, 8, 9]. Furthermore, individuals with INOCA frequently encounter substantial physical restrictions, frequent hospitalizations for chest pain, and a diminished quality of life [10, 11]. However, they often lack appropriate medical management due to inadequate diagnosis and prognostic evaluation [12]. Multiple studies have demonstrated that coronary microvascular dysfunction (CMD), coronary vasospasm, or both are the main endotypes of INOCA and are related to MACE [5]. Unfortunately, few cardiac catheterization laboratories stock specialized coronary wires to evaluate CMD or conduct provocative vasospasm testing. Thus, it is crucial to explore widely applicable indicators for assessing myocardial ischemia and prognosis in patients with INOCA.

The triglyceride glucose (TyG) index, calculated from fasting blood glucose (FBG) and triglyceride (TG) levels, is regarded as an easily accessible surrogate marker for insulin resistance (IR) [13, 14]. It is practical to measure in primary care settings with laboratory capacity. As a synthetic parameter of FBG and TG in individuals, the TyG index has been shown to be associated with an increased risk of cardiovascular diseases (CVD), including atherosclerosis, CAD, and myocardial infarction (MI) [15–18]. Additionally, several studies have demonstrated the predictive role of the TyG index with regard to mortality and adverse cardiovascular outcomes [19–21]. While there is considerable heterogeneity in INOCA patients, there is evidence supporting an association between diabetes or chronic hyperglycemia and the onset of INOCA, which subsequently increases the risk of adverse prognosis [22–24]. However, it remains unclear whether the TyG index, as a potential cardiovascular event marker, is related to the occurrence of myocardial ischemia and adverse prognosis in INOCA patients.

Therefore, the primary objective of this study was to evaluate the correlation between the TyG index and myocardial ischemia based on myocardial perfusion imaging (MPI) in INOCA patients. Additionally, we sought to explore the potential impact of the TyG index in assessing the prognosis risk associated with INOCA, aiming to enhance the convenience and efficiency of managing these patients in clinical settings.

## Methods

### Study population

This retrospective observational study recruited all consecutive INOCA patients who underwent CAG and stress-rest MPI with D-SPECT within 3 months at Shanghai Tenth People’s Hospital from February 2017 to June 2019.

We included aged > 18 years old patients who were diagnosed with myocardial ischemia based on cardiovascular risk profiles, clinical ischemic symptoms and/or signs, and electrocardiogram (ECG) findings and whose coronary stenosis < 50% (including 0% stenosis) in all epicardial arteries and major branch arteries > 2.0 mm in diameter confirmed by CAG. The exclusion criteria included: (1) Obstructive CAD, that is, any naive coronary artery stenosis ≥ 50%; (2) History of percutaneous coronary interventions (PCI), coronary artery bypass graft (CABG), or MI; (3) hemodynamic instability; (4) heart failure; (5) severe heart disease requiring surgical operation; (6) serious concomitant hepatic or renal insufficiency; (7) malignancy or other severe medical illnesses with short, expected survival time;

Our study conformed to the Helsinki Declaration and was approved by the Ethics Committee of Shanghai Tenth People’s Hospital (ethical number: SHSY-IEC-5.0/23K112/P01). Informed consent was obtained from all participants.

### Data collection and definitions

Cardiovascular risk factors, demographics, medical history, laboratory indices, electrocardiogram, echocardiography information, medication use, and the data of CAG were collected in detail from medical files. Blood samples for analyzing FBG, hemoglobin A1c (HbA1c), total cholesterol (TC), TG, low-density lipoprotein-C (LDL-C), high-density lipoprotein-cholesterol (HDL-C), serum creatinine, C-reactive protein (CRP) levels, cardiac troponin T (cTnT), creatine kinase-MB (CK-MB), myoglobin (MYO), N-terminal pro-brain natriuretic peptide (NT-proBNP) were obtained after fasting for more than 8 h.

DM was diagnosed when random plasma glucose ≥ 11.1 mmol/L, FBG ≥ 7.0 mmol/L, 2‑hour plasma glucose ≥ 11.1 mmol/L after OGTT, or HbA1c ≥ 6.5% [25]. The hypertension diagnosis was based on systolic blood pressure (SBP) ≥ 140 mmHg or diastolic blood pressure (DBP) ≥ 90 mmHg at rest or on antihypertensive treatments. Body mass index (BMI) was computed as weight (kg) /height^2^ (m^2^).

The TyG index was calculated with the following equation: TyG index = Ln [fasting TG (mg/dL) × FBG (mg/dL)/2] [26]. INOCA Patients were divided into three groups (T1/T2/T3) based on their TyG index tertiles.

### D-SPECT examination and analysis

Myocardial ischemia was evaluated by MPI, which was detected using the D-SPECT cardiac scanner (Spectrum Dynamics, Biosensors, Caesarea, Israel). Details of the detection and analysis of D-SPECT have been described previously [27]. All patients were given a single-day rest/stress imaging protocol with the use of 99mTc-sestamibi (99mTc-MIBI). Stress testing was performed by intravenous adenosine triphosphate (ATP) in our Department of Nuclear Medicine. After injection of 99mTc-MIBI (3 MBq/kg) for about 1 h, the rest image acquisition began, and the acquisition time was 6 min. At 30 min after the rest image acquisition, each patient was injected intravenously with ATP (140 µg/kg/min, for 6 min), followed by an injection of the 99mTc-MIBI (9 MBq/kg) 3 min later. After an interval of 30–45 min, the stress image was collected, and the collection time was 6 min. Finally, we reoriented the images into short-axis, horizontal, and vertical long-axis slices with QPS software (Cedars-Sinai Medical Center, LA, CA).

MPI images were independently analyzed using QPS software by two experienced nuclear cardiologists who were unaware of the patient’s characteristics and outcomes. The scores were calculated according to the 17-segment model and the five-point scale (“0, 1, 2, 3, 4” in turn stands for “normal, equivocal, moderate, severe, absence tracer uptake “) [28]. The summed rest score (SRS) was obtained by adding all defects in the rest images, and the summed stress score (SSS) was obtained by adding all defects in the stress images. The difference between SRS and SSS calculated the summed difference score (SDS). In addition, we calculated the stress total perfusion defect (TPD). Abnormal MPI for myocardial ischemia in individual coronary territories was defined as SSS ≥ 4 and SDS ≥ 2 [29, 30]. QPS software was also used to measure left ventricular functions such as end-diastolic volume (EDV), end-systolic volume (ESV), transient ischemic dilation (TID), peak ejection rate (PER), and peak filling rate (PFR).

### Follow-up and endpoints

Follow-up data was collected by trained cardiologists through reviewing medical records, outpatient visits, and making phone calls. In our study, the median (interquartile range) follow-up duration was 35 (31–37) months. The clinical endpoint of our study was MACE, which was defined as cardiovascular death, nonfatal MI, heart failure, angina-related rehospitalization, nonfatal stroke, and ischemia-driven revascularization. Cardiovascular death was the death caused by MI, severe arrhythmia, heart failure, or other cardiovascular diseases. Nonfatal MI was defined if they had (1) positive cardiac biomarkers and (2) either typical symptoms of ischemia and/or dynamic ECG changes [31]. Heart failure was diagnosed following previous guidelines [32]. Angina-related rehospitalization refers to the chest pain or dynamic ECG changes associated with chest pain but normal cardiac biomarkers, and it is characterized by new onset angina, rest symptoms, or increasing severity or duration of the earlier stable angina symptoms. The diagnosis of nonfatal stroke was confirmed according to imaging examination and clinical symptoms or signs. Ischemia-driven revascularization was the coronary revascularization owing to a positive test for myocardial ischemia or clinical deterioration.

### Statistical analysis

The statistical analysis of this study was performed using the Statistical Package for Social Sciences (SPSS) v.22. and GraphPad software 8.0.1. The mean ± standard deviation with a normal distribution and medians (interquartile range) with non-normal distribution were used for continuous variables. The t-test or ANOVA was used to compare continuous variables with a normal distribution between groups, and the Mann-Whitney U-test or Kruskal-Wallis test was used to analyze intergroup comparisons of continuous variables with a non-normal distribution. Categorical variables were presented as counts and percentages (%) and compared using Pearson’s chi-squared (χ2) or Fisher’s exact test. Logistic regression analysis evaluated the association between abnormal MPI and clinical risk factors in INOCA patients. The correlation between the TyG index and scores of myocardial perfusion was performed by Pearson or Spearman correlation analysis. The MACE-free survival rates were evaluated with Kaplan–Meier survival curves and compared using the log-rank test. Cox proportional regression analysis determined the relationship between the TyG index and clinical outcome in INOCA patients. The multivariate analysis included the univariate predictors with *P* < 0.10 in logistic regression and Cox proportional regression. We excluded the highly correlated variables when we performed multivariate regression analysis. All analysis was conducted two-sided, and the P-value < 0.05 was considered statistically significant.

## Results

### Baseline characteristics

**Fig. 1:**
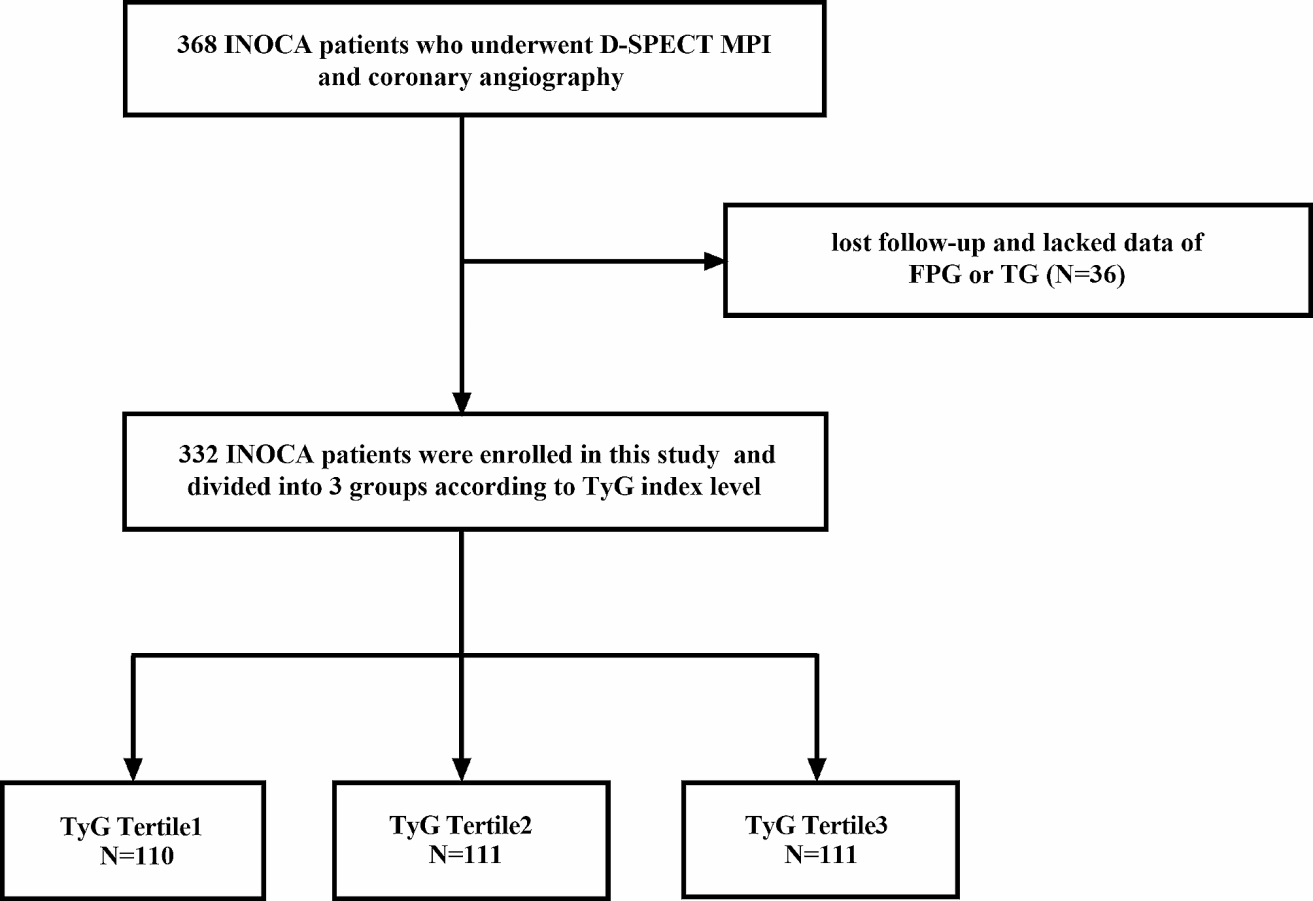
Flow diagram of study patients. *INOCA* ischemia with non-obstructive coronary artery disease, *MPI* myocardial perfusion imaging, *FPG* fasting plasma glucose, *TG* triglyceride, *TyG index* triglyceride-glucose index

A total of 368 INOCA patients who underwent D-SPECT and CAG were included, in which 36 patients lost to follow-up or missed important data. Finally, 332 patients were enrolled in our study (Fig. 1).

Clinical characteristics of the INOCA patients stratified by the TyG index are shown in Table 1. The INOCA patients in the T3 group tended to have diabetes, dyslipidemia, lower HDL-C, LVEF, and higher BMI, TG, FBG, HbA1c, TC, LDL-C, and beta-blocker use.

**Table 1:** Clinical characteristics of the INOCA patients stratified by TyG index

	TyG index level			P value
	T1(*N* = 110)	T2(*N* = 111)	T3(*N* = 111)	
**General characteristics**				
Age (years)	62.03 ± 10.01	59.75 ± 10.20	62.08 ± 9.43	0.135
Male, n (%)	48 (43.6)	49 (44.1)	36 (32.4)	0.132
BMI (kg/m^2^)	23.70 ± 3.19	25.33 ± 3.50	25.43 ± 2.95	< 0.001
Heart rate (beats per minute)	76.14 ± 12.91	77.40 ± 11.91	77.80 ± 10.74	0.555
**Comorbidities**				
Atrial fibrillation, n (%)	9 (8.2)	6 (5.4)	5 (4.5)	0.489
Smoking, n (%)	20 (18.2)	27 (24.3)	19(17.1)	0.349
Drinking, n (%)	9 (8.2)	10 (9.0)	6 (5.4)	0.567
Hypertension, n (%)	54 (49.1)	54 (48.6)	60 (54.1)	0.671
Diabetes, n (%)	15 (13.6)	16 (14.4)	38(34.2)	< 0.001
Dyslipidemia, n (%)	10 (9.1)	23 (20.7)	74 (66.7)	< 0.001
CKD, n (%)	6 (5.5)	2 (1.8)	8 (7.2)	0.159
COPD, n (%)	11 (10.0)	9 (8.1)	7 (6.3)	0.604
**Angiographic characteristics**				
Normal vessels ^a^, n (%)	58 (52.7)	55 (49.5)	54 (48.6)	0.817
Vessel with any stenosis ^b^, n (%)	52 (47.3)	56 (50.5)	57 (51.4)	0.817
**Laboratory values**				
TyG index	8.14(7.86, 8.25)	8.59(8.48, 8.77)	9.17(9.03, 9.50)	< 0.001
TG (mmol/L)	0.87(0.69, 0.96)	1.34(1.19, 1.55)	2.16(1.88, 2.65)	< 0.001
FBG (mmol/L)	4.80(4.40, 5.20)	5.10(4.70, 5.50)	5.50(5.00, 6.30)	< 0.001
HbA1c (%)	5.70(5.50, 6.00)	5.70(5.50, 5.90)	6.00(5.70, 6.70)	< 0.001
CRP (mg/L)	3.02(0.69, 3.23)	3.02(1.86, 3.27)	3.02(1.23, 3.23)	0.383
Serum creatinine (µmol/L)	66.80(58.48, 78.65)	68.00(58.50, 75.55)	69.25(58.50, 78.83)	0.814
TC (mmol/L)	3.86 ± 0.96	4.22 ± 0.98	4.30 ± 1.06	0.003
LDL-C (mmol/L)	2.07 ± 0.83	2.41 ± 0.83	2.56 ± 1.00	< 0.001
HDL-C (mmol/L)	1.28(1.11, 1.51)	1.13(0.99, 1.37)	1.02(0.89, 1.18)	< 0.001
cTnT (ng/mL)	0.007(0.004, 0.010)	0.005(0.004, 0.008)	0.007(0.004, 0.010)	0.099
CK-MB (ng/mL)	1.26(0.95, 1.70)	1.18(0.88, 1.85)	1.25(0.94, 1.78)	0.631
MYO (ng/mL)	23.50(21.00, 37.38)	22.03(21.00, 32.04)	23.38(21.00, 30.24)	0.590
NT-proBNP (pg/mL)	75.07(35.50,139.00)	61.92(27.18, 123.45)	46.10(22.59, 90.74)	0.063
LVEF (%)	65.00(62.00,66.00)	63.00(62.00, 65.50)	63.00(60.00, 65.00)	0.007
**Cardiovascular medical therapy**				
Aspirin, n (%)	44 (40.0)	53 (47.7)	54 (48.6)	0.366
Clopidogrel, n (%)	26 (23.6)	29 (26.1)	34 (30.6)	0.492
Statin, n (%)	77 (70.0)	85 (76.6)	84 (75.7)	0.482
ACEI/ARB, n (%)	35 (31.8)	28 (25.2)	44 (39.6)	0.071
Beta blocker, n (%)	41 (37.3)	31 (27.9)	50 (45.0)	0.030
CCB, n (%)	40 (36.4)	48 (43.2)	38 (34.2)	0.352

*INOCA* ischemia with non-obstructive coronary artery disease, *TyG index* triglyceride-glucose index, *BMI* body mass index, *CKD* chronic kidney disease, *COPD* chronic obstructive pulmonary disease, *TG* triglyceride, *FBG* fasting blood glucose, *HbA1c* Hemoglobin A1c, *CRP* C-reactive protein, *TC* total cholesterol, *LDL-C* low-density lipoprotein-cholesterol, *HDL-C* high-density lipoprotein-cholesterol, *cTnT* cardiac troponin T, *CK-MB* creatine kinase-MB, *MYO* myoglobin, *NT-proBNP* N-terminal pro-brain natriuretic peptide, *LVEF* left ventricular ejection fraction, *ACEI/ARB* angiotensin-converting-enzyme inhibitors/angiotensin receptor blockers, *CCB* calcium channel blocker

^a^Vessels with 0% stenosis

^b^Vessels with 0–50% stenosis

**Fig. 2:**
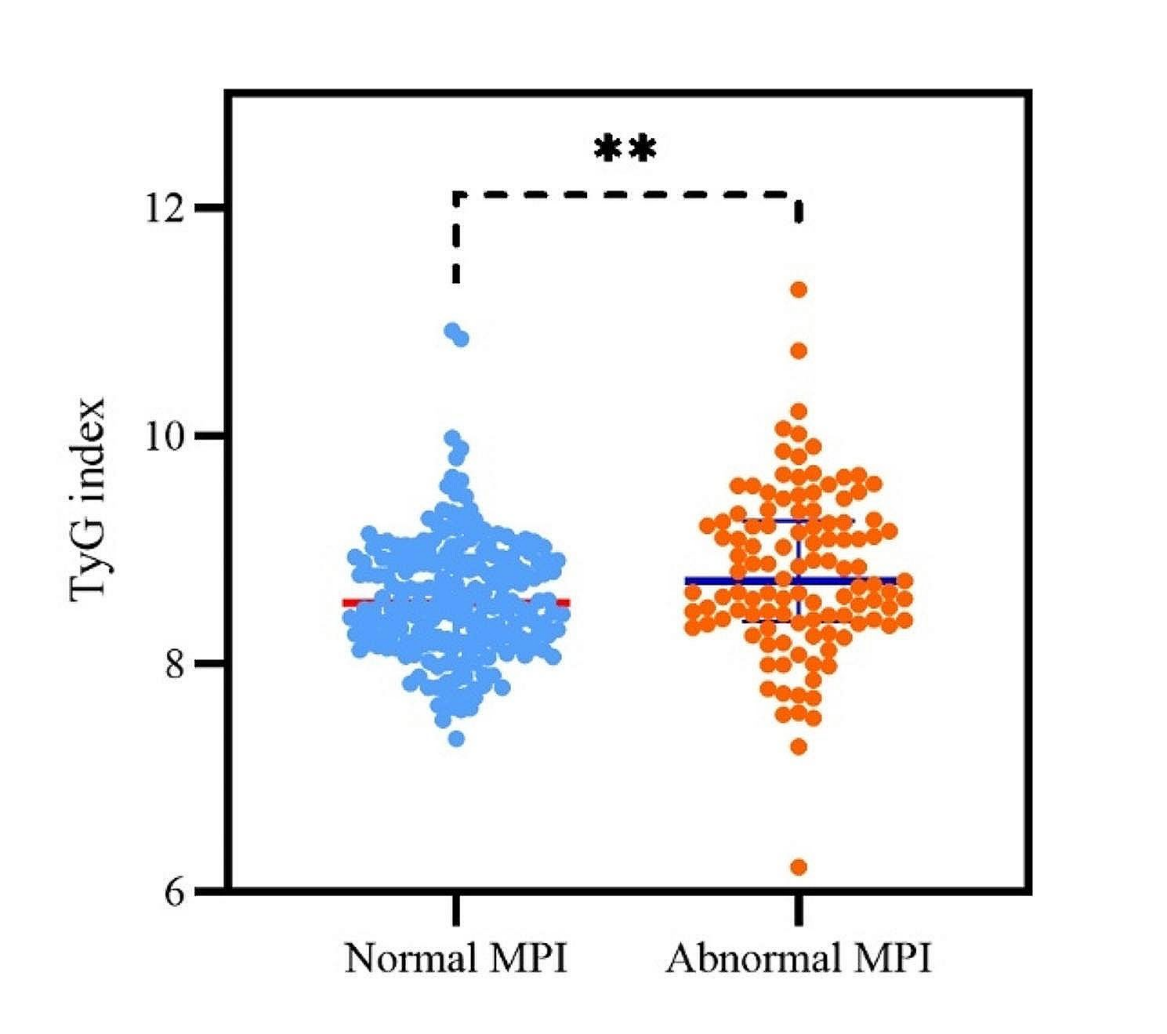
Comparison of TyG index in INOCA patients with normal MPI and abnormal MPI. *TyG index* triglyceride-glucose index, *INOCA* ischemia with non-obstructive coronary artery disease, *MPI* myocardial perfusion imaging, **P < 0.01

Table 2 shows the D-SPECT data of INOCA patients stratified by TyG index. Of 332 INOCA patients, 113 (34.0%) had abnormal MPI. INOCA patients with higher TyG index had a higher rate of abnormal MPI (25.5% vs. 32.4% vs. 44.1%; *p* = 0.012). Compared to the lower TyG index groups, the T3 group had significantly higher SSS, SRS, stress TPD, and lower resting PFR. INOCA patients with abnormal MPI had higher TyG index than those with normal MPI [8.73 (8.37,9.25) vs. 8.54 (8.21,8.93); *P* = 0.001] (Fig. 2).

**Table 2:** D-SPECT data of INOCA patients stratified by TyG index

	TyG index level			P value
	T1(*N* = 110)	T2(*N* = 111)	T3(*N* = 111)	
Abnormal MPI, n (%)	28 (25.5)	36 (32.4)	49 (44.1)	0.012
Summed stress score	1(0,3)	1(0,3)	2(0,5)	0.014
Summed rest score	0(0,0)	0(0,1)	0(0,2)	0.003
Summed difference score	1(0,2)	1(0,2)	2(0,3)	0.094
Stress TPD (%)	1(1,3)	2(0,3)	2(1,6)	0.013
Perfusion defect location				
Anterior, n (%)	24 (21.8)	39 (35.1)	47 (42.3)	0.005
Lateral, n (%)	30 (27.3)	26 (23.4)	45 (40.5)	0.015
Inferior or posterior, n (%)	32 (29.1)	21 (18.9)	26 (23.4)	0.205
Septum, n (%)	29 (26.4)	26 (23.4)	41 (36.9)	0.065
Apex, n (%)	15 (13.6)	14 (12.6)	17 (15.3)	0.841
Left ventricular functional parameters				
Stress EDV (mL)	67.00(54.00,80.00)	68.50(56.50,89.25)	66.00(53.75,80.50)	0.430
Stress ESV (mL)	21.00(16.00,29.00)	24.00(17.75,32.25)	22.50(15.00,29.25)	0.240
Stress PER (- EDV/s)	3.54 ± 0.60	3.45 ± 0.75	3.53 ± 0.95	0.636
Stress PFR (EDV/s)	2.59 ± 0.73	2.60 ± 2.21	2.29 ± 0.83	0.191
Rest EDV (mL)	62.00(49.00,74.00)	63.00(54.75,79.00)	64.00(48.00,79.00)	0.276
Rest ESV (mL)	18.00(11.50,25.00)	21.00(14.00,29.00)	20.00(12.00,29.00)	0.098
Rest PER (- EDV/s)	3.74 ± 0.71	3.52 ± 0.67	3.59 ± 1.03	0.064
Rest PFR (EDV/s)	2.77 ± 0.85	2.46 ± 0.67	2.36 ± 0.79	< 0.001
TID	1.11 ± 0.14	1.09 ± 0.14	1.09 ± 0.14	0.501

*INOCA* ischemia with non-obstructive coronary artery disease, *TyG index* triglyceride-glucose index, *MPI* myocardial perfusion imaging, *TPD* total perfusion defects, *EDV* end-diastolic volume, *ESV* end-systolic volume, *PER* peak ejection rate, *PFR* peak filling rate, *TID* transient ischemic dilation

### Association between TyG index and MPI in INOCA patients

**Fig. 3:**
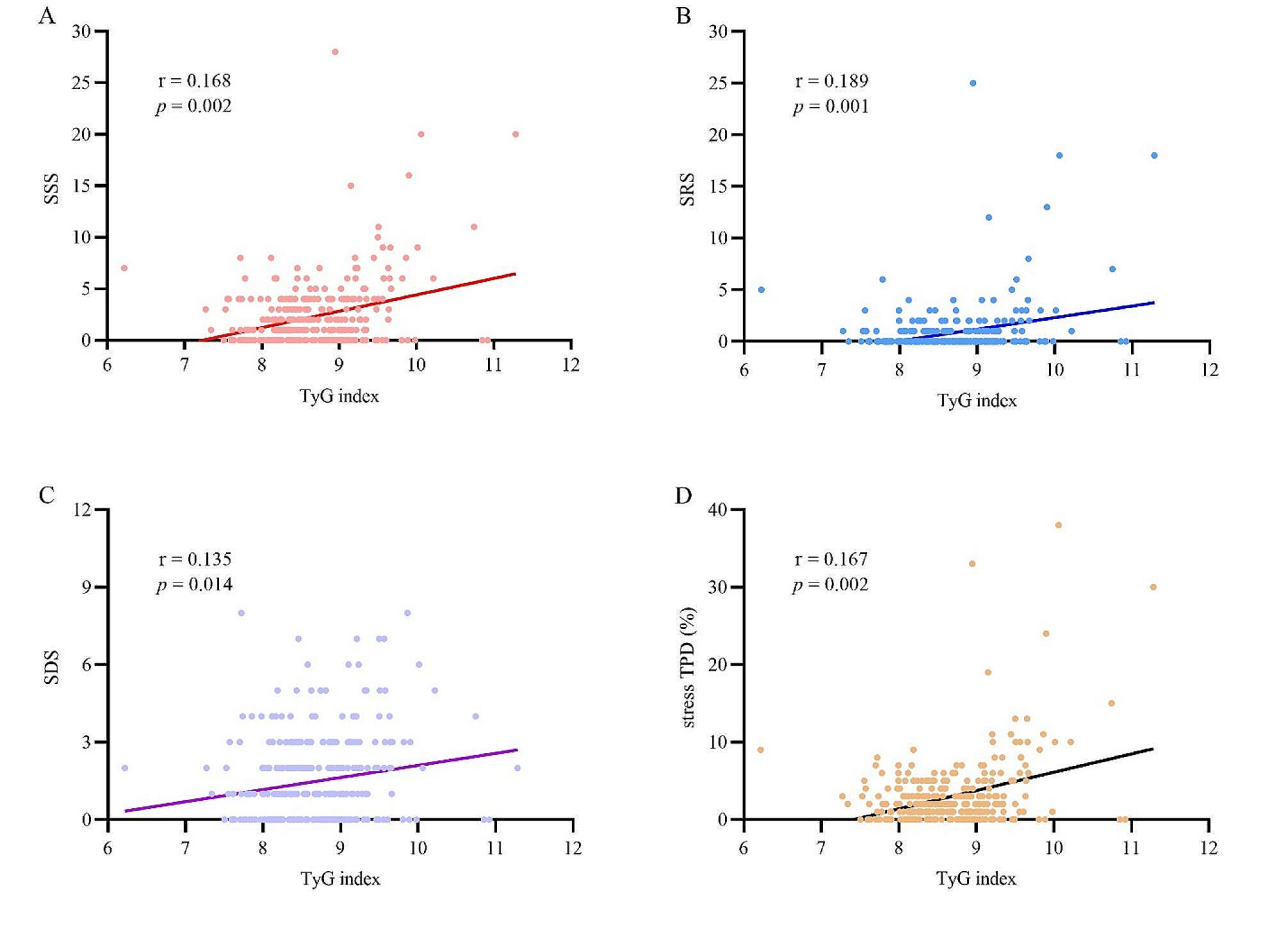
Correlation between TyG index and SSS (**A**), SRS (**B**), SDS (**C**) and stress TPD (**D**). *TyG index* triglyceride-glucose index, *SSS* summed stress score, *SRS* summed rest score, *SDS* summed difference score, *TPD* total perfusion defect

Table 3 presents the association between clinical risk factors and abnormal MPI in INOCA patients. Univariable logistic regression analysis showed that females, high TyG index, TG, and low LVEF were significantly correlated with abnormal MPI in INOCA patients, and the T3 group had a 2.315-fold risk of abnormal MPI compared to the T1 group (OR, 2.315; 95% CI 1.309–4.091; *P* = 0.004). In the multivariate analysis, a high TyG index was still significantly associated with abnormal MPI in INOCA patients (OR, 1.901; 95% CI, 1.045–3.458; *P* = 0.035). In addition, gender also remained correlated with abnormal MPI. The correlation between the TyG index and scores of myocardial perfusions, such as SSS, SRS, SDS, and stress TPD, was examined. The results indicated that the TyG index correlated well with SSS, SRS, SDS, and stress TPD among INOCA patients (*r* = 0.168, *r* = 0.189, *r* = 0.135, and *r* = 0.167, respectively) (Fig. 3).

**Table 3:** Association between abnormal MPI and clinical risk factors in INOCA patients

	Univariate analysis OR (95% CI)	P value	Multivariate analysis OR (95% CI)	P value
Age	0.995 (0.973–1.018)	0.674		
Male	0.588 (0.365–0.948)	0.029	0.589 (0.357–0.972)	0.038
BMI	1.010 (0.943–1.081)	0.784		
Heart rate	1.000 (0.981–1.019)	0.992		
Atrial fibrillation	1.046 (0.405–2.701)	0.925		
Smoking	0.883 (0.496–1.571)	0.671		
Drinking	1.320 (0.573–3.042)	0.514		
Hypertension	1.296 (0.822–2.044)	0.265		
Diabetes	0.961 (0.548–1.685)	0.890		
Coronary artery stenosis	0.718 (0.455–1.133)	0.154		
TyG index	1.833 (1.245–2.698)	0.002	-	
TyG index tertiles				
T1	Reference		Reference	
T2	1.406 (0.783–2.523)	0.254	1.316 (0.723–2.398)	0.369
T3	2.315 (1.309–4.091)	0.004	1.901 (1.045–3.458)	0.035
TG	1.577 (1.219–2.041)	0.001	-	
FBG	1.064 (0.897–1.263)	0.476		
HbA1c	1.114 (0.852–1.456)	0.431		
CRP	1.001 (0.977–1.026)	0.922		
Serum creatinine	0.995 (0.982–1.007)	0.397		
TC	0.904 (0.721–1.133)	0.381		
LDL-C	0.817 (0.633–1.055)	0.121		
HDL-C	1.362 (0.673–2.755)	0.391		
cTnT, per 0.1 ng/mL	1.002 (0.867–1.159)	0.978		
CK-MB	0.989 (0.951–1.029)	0.580		
MYO	0.988 (0.972–1.005)	0.159		
NT-proBNP, per 10 pg/mL	1.000 (0.998–1.001)	0.716		
LVEF	0.958 (0.921–0.997)	0.037	0.964 (0.924–1.004)	0.079
Aspirin	0.927 (0.587–1.464)	0.746		
Clopidogrel	1.371 (0.829–2.268)	0.219		
Statin	1.175 (0.695–1.986)	0.548		
ACEI/ARB	1.243 (0.769–2.011)	0.375		
Beta blocker	1.089 (0.681–1.741)	0.723		
CCB	1.193 (0.749-1.900)	0.457		

*MPI* myocardial perfusion imaging, *INOCA* ischemia with non-obstructive coronary artery disease, *BMI* body mass index, *TyG index* triglyceride-glucose index, *TG* triglyceride, *FBG* fasting blood glucose, *HbA1c* Hemoglobin A1c, *CRP* C-reactive protein, *TC* total cholesterol, *LDL-C* low-density lipoprotein-cholesterol, *HDL-C* high-density lipoprotein-cholesterol, *cTnT* cardiac troponin T, *CK-MB* creatine kinase-MB, *MYO* myoglobin, *NT-proBNP* N-terminal pro-brain natriuretic peptide, *LVEF* left ventricular ejection fraction, *ACEI/ARB* angiotensin-converting-enzyme inhibitors/angiotensin receptor blockers, *CCB* calcium channel blocker, *OR* odds ratio, *CI* confidence interval

### Outcome of INOCA patients according to the TyG index

**Fig. 4:**
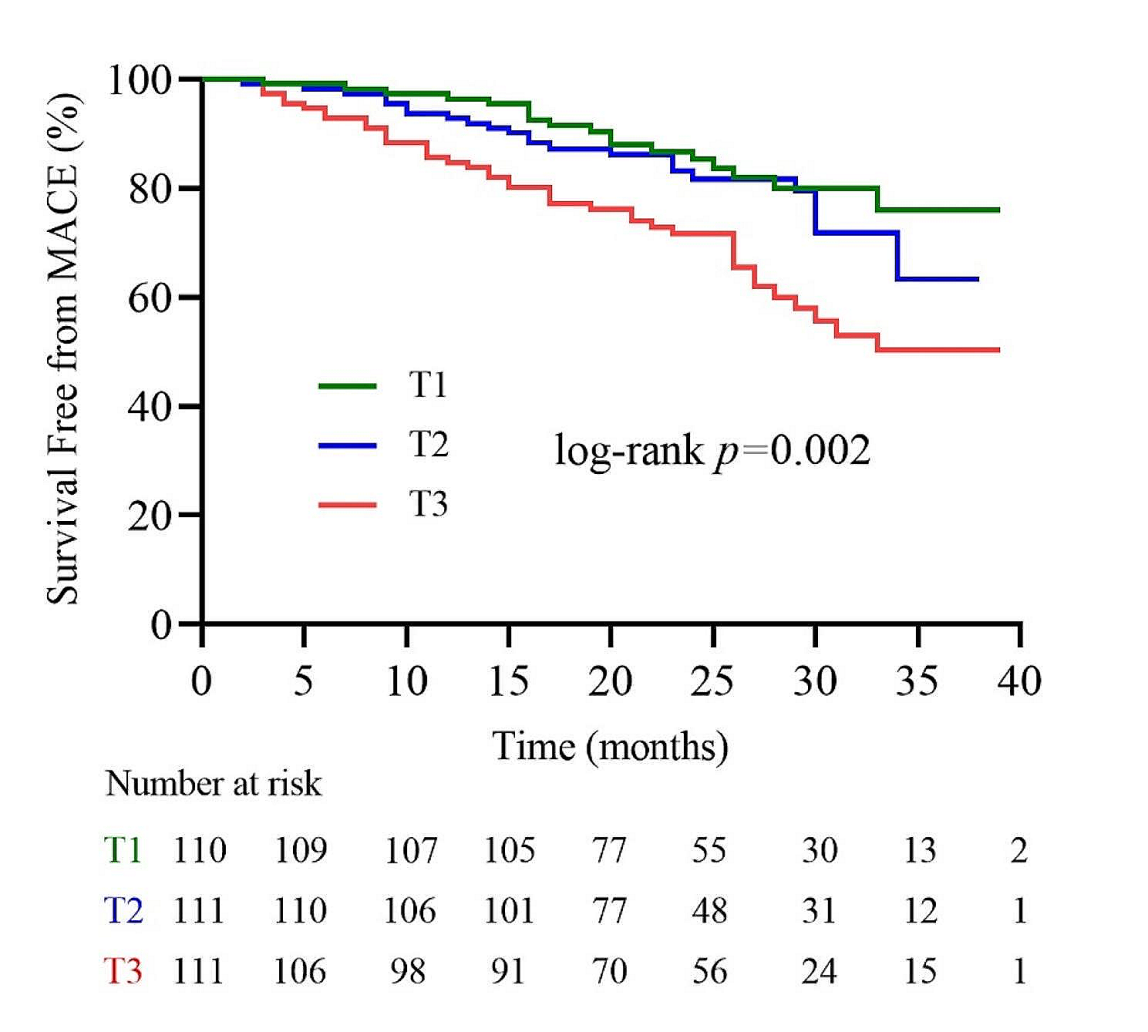
Kaplan-Meier survival curve for MACE based on TyG index tertiles. *MACE* major adverse cardiovascular events, *TyG index* triglyceride-glucose index, *T1* TyG index tertile 1, *T2* TyG index tertile 2, *T3* TyG index tertile 3

To explore the influence of the TyG index on the clinical outcome of INOCA patients, we compared the outcomes of patients with different TyG index levels. Over the follow-up period, 83 (25%) MACE were recorded among the INOCA patients, including 1 cardiovascular death, 2 nonfatal MI, 11 heart failure, 57 Angina-related rehospitalizations, 10 nonfatal stroke, and 2 ischemia-driven revascularizations. A higher incidence of MACE was observed in the INOCA patients with higher TyG index (T3 vs. T2 vs. T1: 36.9% vs. 21.6% vs. 16.4%, respectively; *p* = 0.001) (Table 4). In Fig. 4, Kaplan-Meier curves showed that the patients in the T3 group had the highest risk of MACE compared to those in the T1 and T2 groups (log-rank *P* = 0.002).

**Table 4:** INOCA patients’ outcomes according to TyG index

	TyG index level			P-value
	T1 (*n* = 110)	T2 (*n* = 111)	T3 (*n* = 111)	
**MACE**	18(16.4)	24(21.6)	41(36.9)	0.001
Cardiovascular death	0	0	1(0.9)	1.000
Nonfatal MI	0	1(0.9)	1(0.9)	1.000
Heart failure	1(0.9)	3(2.7)	7(6.3)	0.091
Angina-related rehospitalization	14(12.7)	17(15.3)	26(23.4)	0.089
Nonfatal stroke	3(2.7)	3(2.7)	4(3.6)	1.000
Ischemia-driven revascularization	0	0	2(1.8)	0.331

*INOCA* ischemia with non-obstructive coronary artery disease, *TyG index* triglyceride-glucose index, *MACE* major adverse cardiovascular event, *MI* myocardial infarction

### Association between TyG index and clinical outcomes in INOCA patients

The Cox regression analysis for MACE of INOCA patients is shown in Table 5. In the univariate Cox regression analysis, a high TyG index (T3 group) was significantly associated with the risk of MACE (HR, 2.478; 95% CI 1.424–4.314; *P* = 0.001). Gender, BMI, hypertension, serum creatinine, LVEF, and abnormal MPI were also the predictive factors of MACE. After adjusting for the univariate predictors with *P* < 0.10, the T3 group still had a 2.338-fold risk of MACE compared with the T1 group (adjusted HR, 2.338; 95% CI 1.253–4.364, *P* = 0.008), in addition, hypertension and abnormal MPI also remained associated with the risk of MACE.

**Table 5:** Cox regression analysis for MACE of INOCA patients

	Univariate analysis HR (95% CI)	P value	Multivariate analysis HR (95% CI)	P value
Age	1.020 (0.996–1.044)	0.105		
Male	1.592 (1.034–2.451)	0.035	1.174 (0.702–1.965)	0.541
BMI	1.081 (1.010–1.157)	0.024	1.034 (0.957–1.118)	0.392
Heart rate	0.999 (0.981–1.017)	0.874		
Atrial fibrillation	1.727 (0.833–3.583)	0.142		
Smoking	0.846 (0.484–1.481)	0.558		
Drinking	1.182 (0.545–2.564)	0.672		
Hypertension	2.068 (1.316–3.249)	0.002	1.653 (1.018–2.683)	0.042
Diabetes	1.429 (0.877–2.328)	0.152		
Coronary artery stenosis	1.472 (0.952–2.276)	0.082	1.498 (0.947–2.370)	0.084
TyG index	1.824 (1.322–2.517)	< 0.001	-	
TyG index tertiles				
T1	Reference		Reference	
T2	1.360 (0.738–2.505)	0.325	1.265 (0.657–2.436)	0.482
T3	2.478 (1.424–4.314)	0.001	2.338 (1.253–4.364)	0.008
TG	1.338 (1.170–1.529)	< 0.001	-	
FBG	1.097 (0.960–1.253)	0.175		
HbA1c	1.130 (0.901–1.417)	0.291		
CRP	1.010 (0.991–1.029)	0.297		
Serum creatinine	1.008 (1.002–1.014)	0.014	1.009 (0.995–1.022)	0.202
TC	0.829 (0.664–1.033)	0.095	0.836 (0.665–1.051)	0.125
LDL-C	0.968 (0.761–1.232)	0.793		
HDL-C	0.701 (0.348–1.412)	0.320		
cTnT, per 0.1 ng/mL	0.729 (0.372–1.426)	0.356		
CK-MB	0.964 (0.893–1.041)	0.349		
MYO	1.000 (0.996–1.004)	0.907		
NT-proBNP, per 10 pg/mL	0.999 (0.996–1.002)	0.567		
LVEF	0.957 (0.930–0.986)	0.003	0.979 (0.948–1.011)	0.190
Abnormal MPI	1.837 (1.192–2.829)	0.006	1.642 (1.022–2.638)	0.040
Aspirin	0.720 (0.463–1.118)	0.143		
Clopidogrel	0.862 (0.525–1.414)	0.555		
Statin	0.751 (0.467–1.208)	0.237		
ACEI/ARB	1.321 (0.847–2.062)	0.220		
Beta blocker	1.220 (0.789–1.886)	0.372		
CCB	1.155 (0.747–1.785)	0.518		

*MACE* major adverse cardiovascular event, *INOCA* ischemia with non-obstructive coronary artery disease, *BMI* body mass index, *TyG index* triglyceride-glucose index, *TG* triglyceride, *FBG* fasting blood glucose, *HbA1c* Hemoglobin A1c, *CRP* C-reactive protein, *TC* total cholesterol, *LDL-C* low-density lipoprotein-cholesterol, *HDL-C* high-density lipoprotein-cholesterol, *cTnT* cardiac troponin T, *CK-MB* creatine kinase-MB, *MYO* myoglobin, *NT-proBNP* N-terminal pro-brain natriuretic peptide, *LVEF* left ventricular ejection fraction, *MPI* myocardial perfusion imaging, *ACEI/ARB* angiotensin-converting-enzyme inhibitors/angiotensin receptor blockers, *CCB* calcium channel blocker, *HR* hazard ratio, *CI* confidence interval

## Discussions

The main findings of our investigation were: (1) A notable association was observed between high TyG index and abnormal MPI in patients with INOCA. (2) The incidence of MACE was significantly elevated in the high TyG compared to the low TyG index. (3) The TyG index emerged as an independent prognostic factor linked to an unfavorable prognosis in INOCA patients. Our research findings highlight the prognostic relevance of the TyG index in predicting both myocardial ischemia and the occurrence of MACE in individuals with INOCA.

INOCA refers to individuals who manifest symptoms and signs indicative of myocardial ischemia but lack clear evidence of coronary artery obstruction [5]. Initially, INOCA was not deemed a pathologically significant condition; however, recent research has indicated that individuals with INOCA exhibit a comparable risk of MACE while experiencing a lower quality of life in comparison to patients with obstructive CAD [33, 34]. Furthermore, a meta-analysis encompassing patients diagnosed with INOCA revealed elevated rates of all-cause mortality and nonfatal myocardial infarction in comparison to the general population [35]. Consequently, the precise stratification of risk among patients with INOCA and the timely identification of associated risk factors are paramount in clinical settings.

As of now, there is compelling evidence highlighting a close association between abnormal myocardial perfusion and the prognosis of individuals diagnosed with INOCA [24, 27]. Many studies have explored the significance of radionuclide myocardial perfusion imaging in assessing the risk of patients with INOCA. Our previous investigation demonstrated that INOCA patients exhibiting perfusion defects indicative of abnormal MPI correlate with an unfavorable prognosis [27]. Similarly, Zampella et al. observed that abnormal MPI effectively identifies INOCA patients at a heightened risk of cardiac events [36].

Traditional risk factors such as diabetes mellitus, hypertension, age, and smoking may all be associated with INOCA and CMD [3, 5]. In particular, individuals with diabetes are more susceptible to endothelial dysfunction, inflammation, and oxidative stress, which are significant pathophysiological drivers involved in INOCA [37]. Studies have demonstrated reduced myocardial flow reserve and worse microvascular dysfunction in diabetic patients [22, 38, 39]. Equally important, diabetes or hyperglycemia have been shown to predict worse outcomes in INOCA patients, including increased all-cause mortality [24, 40, 41]. However, some studies have indicated that diabetes is not commonly found among INOCA patients, and some patients without traditional risk factors may still develop INOCA and CMD [42]. This highlights the importance of identifying novel risk factors for INOCA in the general population prior to the onset of diabetes.

IR often manifests before the presence of traditional risk factors, and IR serves as a crucial intermediate process of metabolic disorders, diabetes mellitus, and coronary artery disease [43, 44]. It represents a central aspect of disruptions in blood glucose homeostasis and has been substantiated as a robust risk factor for CVD [45, 46]. Beyond its role in the onset of CVD, IR significantly influences the prognostication of individuals with these conditions [45, 47, 48]. Consequently, the detection of IR holds crucial clinical significance in stratifying the risk of CVD.

Currently, there are few methods for detecting IR. The euglycemic insulin clamp and intravenous glucose tolerance testing is accurate but expensive and invasive. As for the homeostasis model assessment-estimated insulin resistance (HOMA-IR) index, there is very little value in patients treated with insulin or those without functioning beta cells, although it’s currently widely used [49]. Notably, the TyG index, an emerging marker of IR, has recently been advocated as a convenient and reliable substitute for assessing IR [50, 51]. Compared with traditional methods for detecting IR, the TyG index is a convenient, low-cost, and reliable surrogate, regardless of insulin treatment [49, 52]. Moreover, the TyG index has garnered attention recently due to its potential prognostic significance across various CVDs [49, 53]. Liu et al. observed that an elevated TyG index was potentially linked to a heightened incidence of CAD and MI in the general population [21]. Numerous studies have also identified a positive correlation between the TyG index and the severity of CAD. This finding suggests a potential association between TyG and the severity of the disease and the presence of ischemic symptoms [54, 55]. Nevertheless, the association between the TyG index and myocardial ischemia in individuals with INOCA remains unclear. In the current investigation, we observed that individuals with INOCA who belonged to the higher TyG index group (T3) demonstrated a heightened occurrence of abnormal MPI. Furthermore, the highest TyG index was significantly associated with an increased risk of abnormal MPI in INOCA patients. Additionally, we identified a significant correlation between the TyG index and ischemic parameters, including SSS, SRS, SDS, and TPD, in individuals diagnosed with INOCA, suggesting that the TyG index may possess predictive value for potential myocardial ischemia in individuals with INOCA. Prior investigations have suggested that IR may compromise vascular endothelial function [56, 57], potentially contributing to cardiac microcirculatory dysfunction and ultimately culminating in myocardial ischemia. An alternate explanation posits that IR could restrict glucose bioavailability, instigating alterations in fatty acid metabolism. This, in turn, may elevate myocardial oxygen consumption and diminish myocardial compensatory capacity, thereby precipitating myocardial ischemia [58]. Our findings may align with prior research, indicating a possible correlation between the TyG index and myocardial ischemia in individuals with INOCA. The heightened occurrence of abnormal MPI in the higher TyG index group, coupled with significant associations with ischemic parameters, suggests that the TyG index may serve as a predictive marker for potential myocardial ischemia in individuals with INOCA.

The TyG index has demonstrated independent predictive capabilities for cardiac events across diverse patient populations [17, 49, 54, 59–61]. A large retrospective study in China suggested that the TyG index could serve as a valuable marker for risk stratification and prognosis in patients with diabetes and acute coronary syndrome [19]. Additionally, findings from the ACROSS-China registration study indicated that a higher TyG index was correlated with an increased risk of stroke recurrence and mortality [62]. However, its specific association with prognosis in individuals diagnosed with INOCA has not been thoroughly elucidated. During the follow-up period, 83 MACEs were recorded in our study. Notably, a higher incidence of MACE was observed in the high TyG group compared to the low TyG group. In the present study, our data was consistent with previous relevant research, which indicated that a higher TyG index is independently associated with an increased incidence of atherosclerotic cardiovascular diseases, CAD, and stroke in the general population [17, 21]. In the adjusted Cox regression analysis, the high TyG group exhibited a substantial association with an increased risk of MACE. The specific mechanisms underlying the association between the TyG index and adverse events in individuals with INOCA remain uncertain. The observed heightened abnormal and significant correlation between the TyG index and ischemic parameters in individuals with INOCA, especially in the higher TyG index group (T3), suggests a potential link between IR and adverse cardiovascular outcomes. IR, reflected by the TyG index, may contribute to microvascular dysfunction, leading to a higher prevalence of abnormal MPI and an increased risk of MACE in INOCA patients. Further studies are warranted to validate these findings and explore the potential link between the TyG index and adverse outcomes in individuals with INOCA.

This study found that the TyG index was closely related to myocardial ischemia and adverse prognosis in INOCA patients. The elevated TyG index was related to myocardial ischemia and an independent predictor of MACE in patients with INCOA. This suggests that the TyG index has broad clinical application prospects in INOCA patients, and the TyG index can be used to stratify the risk of INOCA patients, identify high-risk patients, and provide targeted treatment to improve the clinical outcome of patients. In addition, since the TyG index is an indicator to evaluate IR, whether the treatment for IR can improve the prognosis of INOCA patients is worthy of further exploration.

## Strengths and limitations

The strength of our research lies in its clear demonstration of the prognostic value of the TyG index in individuals with INOCA. By establishing a robust association between the TyG index and both myocardial ischemia and the incidence of MACE, our findings provide a concise and clinically relevant insight into the predictive capabilities of TyG in this specific population. This succinct yet impactful observation has the potential to inform healthcare practices, assisting clinicians in risk assessment and timely interventions for individuals with INOCA, ultimately enhancing patient care and outcomes. Nevertheless, it is essential to acknowledge several limitations in our study. First, being a retrospective and observational analysis of INOCA patients selected for MPI using a D-SPECT camera and CAG, inherent biases associated with such study designs need consideration. Second, being a single-center clinical study, its generalizability to other institutions and different camera designs, such as CZT and conventional camera designs, may be limited. Third, the study’s participants were exclusively Chinese INOCA patients, limiting the direct generalizability of the results to INOCA patients of other races. Despite these limitations, our study suggests that the TyG index might serve as a promising method for risk stratification and prognosis prediction in patients with INOCA, though confirmation through large-sample, prospective, multicenter studies are warranted.

## Conclusion

In conclusion, this study presents the inaugural evidence of a significant association between the TyG index and myocardial ischemia in individuals with INOCA. Furthermore, our findings propose that the TyG index has the potential to serve as a valuable predictor for MACE among INOCA patients. These results imply that the TyG index holds promise as a useful indicator for risk stratification in individuals with INOCA.

## Data Availability

The data analyzed in this study can be obtained from the corresponding author with a reasonable request.

## Abbreviations

INOCA: ischemia and no obstructive coronary artery disease
TyG index: triglyceride-glucose index
MPI: myocardial perfusion imaging
CAD: coronary artery disease
CAG: coronary angiography
MACE: major adverse cardiac events
CMD: coronary microvascular dysfunction
FBG: fasting blood glucose
TG: triglycerides
IR: insulin resistance
CVD: cardiovascular disease
CKD: chronic kidney disease
COPD: chronic obstructive pulmonary disease
MI: myocardial infarction
ECG: electrocardiogram
PCI: percutaneous coronary intervention
CABG: coronary artery bypass graft
LVEF: left ventricular ejection fraction
HbA1c: hemoglobin A1c
TC: total cholesterol
LDL: low-density lipoprotein
HDL: high-density lipoprotein
CRP: C-reactive protein
cTnT: cardiac troponin T
CK-MB: creatine kinase-MB
MYO: myoglobin
NT-proBNP: N-terminal pro-brain natriuretic peptide
SBP: systolic blood pressure
DBP: diastolic blood pressure
BMI: body mass index
SRS: summed rest score
SSS: summed stress score
SDS: summed difference score
TPD: total perfusion defect
EDV: end-diastolic volume
ESV: end-systolic volume
TID: transient ischemic dilation
PER: peak ejection rate
PFR: peak filling rate
